# Surface Roughness Prediction in Ultra-Precision Milling: An Extreme Learning Machine Method with Data Fusion

**DOI:** 10.3390/mi14112016

**Published:** 2023-10-29

**Authors:** Suiyan Shang, Chunjin Wang, Xiaoliang Liang, Chi Fai Cheung, Pai Zheng

**Affiliations:** State Key Laboratory of Ultra-Precision Machining Technology, Department Industrial and Systems Engineering, The Hong Kong Polytechnic University, Hong Kong, China; chunjin.wang@polyu.edu.hk (C.W.); mexiaoliang.liang@polyu.edu.hk (X.L.); pai.zheng@polyu.edu.hk (P.Z.)

**Keywords:** surface roughness prediction, ultra-precision machining, milling, extreme learning machine, feature-level data fusion

## Abstract

This paper pioneers the use of the extreme learning machine (ELM) approach for surface roughness prediction in ultra-precision milling, leveraging the excellent fitting ability with small datasets and the fast learning speed of the extreme learning machine method. By providing abundant machining information, the machining parameters and force signal data are fused on the feature level to further improve ELM prediction accuracy. An ultra-precision milling experiment was designed and conducted to verify our proposed data-fusion-based ELM method. The results show that the ELM with data fusion outperforms other state-of-art methods in surface roughness prediction. It achieves an impressively low mean absolute percentage error of 1.6% while requiring a mere 18 s for model training.

## 1. Introduction

Ultra-precision machining holds great promise for generating surfaces with sub-micrometer form accuracy and surface roughness at the nanometer level, which are widely used in advanced optics and the biomedical, electronic, and aerospace industries [1,2]. As one of the ultra-precision machining technologies, ultra-precision milling can achieve high-level surface quality, which finds extensive applications across various industries, such as the semiconductor and optical fields [3]. Surface quality is physically assessed using precision measuring equipment and instruments, such as the non-contact optical surface profiling system provided by New Zygo NexView. However, traditional physical measurement methods for surface quality detection tend to be time-consuming. To address this issue, an artificial intelligence-based model for surface roughness prediction can be developed on the basis of processing information and machining parameters, through which surface roughness can be generated as soon as the processing is completed. The advancement of machine tool automation has enabled the collection of machining process information by arranging sensors on the machine tool. Thus, the real-time monitoring of the machining conditions and conjecture about surface roughness can be realized without physical metrology operations, which are vital for increasing machining efficiency and guaranteeing machining surface quality [4].

Intelligent surface roughness prediction based on machine learning methods can predict surface roughness through signal data in real time once the prediction model is constructed, which can quickly detect defective workpieces [5]. Classical machine learning algorithms, such as random forest, support vector machine, and artificial neural network [6,7,8], have demonstrated their effectiveness in industrial scenarios. However, deep learning models like Transformer and BERT, commonly applied in speech recognition [9] and natural language processing [10], achieve better prediction accuracy and possess more complex structures and less dependency on expert knowledge [11]. However, it is worth noting that deep learning models require a large-scale dataset to attain optimal performance, which largely limits the application of deep learning models in scenarios where only a limited dataset is available [12]. In ultra-precision machining scenarios, the collection of sensor data and their corresponding real-measured surface roughness data is typically constrained to a limited amount, often several hundreds of samples, which renders most deep learning models unsuitable for this scenario. 

A way to achieve high prediction accuracy while overcoming a limited dataset is a crucial research problem for surface roughness prediction in ultra-precision machining. Most machine learning predictive methods typically train their model based on the backpropagation mechanism, which is time-consuming because network parameters should be updated in repeated iterations [13]. In contrast, ELM has been demonstrated to be effective in many prediction tasks with small datasets because of its fast learning speed, facilitated by the rapid determination of network parameters using Moore–Penrose within several seconds instead of training iteratively with the backpropagation mechanism [14]. Besides a small dataset demand and the quick determination of network parameters, ELM maintains good prediction accuracy at the same time. Cojbasic et al. [15] applied the extreme learning machine to the surface roughness prediction of abrasive water jet machining. However, they only adopted cutting parameters, such as the thickness of the workpiece, abrasive flow rate, and cutting speed, as the model input. To further improve ELM prediction accuracy, an approach is proposed to leverage sensor data that accurately reflects real-time machining conditions by concatenating it with machining parameters for prediction. Specifically, a feature-level fusion technique is employed to seamlessly merge the extracted features from both the sensor data and machining parameters, thus enabling a more comprehensive and robust prediction model.

Considering that research focusing on applying machine learning prediction models to ultra-precision milling has received relatively little attention, this paper presents the use of ELM to develop a surface roughness prediction model for ultra-precision milling that can ensure prediction accuracy and significantly accelerate learning speed. By providing richer machining information, feature-level sensor data and machining parameter fusion can further enhance ELM prediction accuracy. 

## 2. Related Works

Many researchers have been dedicated to researching surface roughness prediction with machine learning methods for decades. One of the most frequently used methods is the artificial neural network (ANN). Zain et al. [16] used ANN techniques for intelligent surface roughness measuring. The experimental model incorporated three input variables, namely cutting speed, feed rate, and rake angle, which were designated as nodes of the artificial neural network (ANN) input layer. Different numbers of nodes in the hidden layer were tested to decide the structure of the ANN. It was found that the 3-1-1 network structure provided the best ANN model. Similarly, Karayel [17] predicted and controlled surface roughness in turning using an ANN with cutting parameters as the input. They are the depth of cut, the cutting speed, and the feed rate. It was found that the feed rate is a dominant parameter, and the effect of depth of cut on surface roughness is not regular. A feed-forward multi-layered neural network was developed, and the network model was trained using the scaled conjugate gradient algorithm. 

Deep learning methods have emerged in recent years in applications in this field. Features extracted in the time domain and frequency domain from original and decomposed signals were used to predict the surface roughness accurately in the grinding process by Guo et al. [18]. The long short-term memory (LSTM) network is applied to forecast ground surface roughness with the input of the grinding force signal, the acceleration signal, and the acoustic emission signal. Another newly emerged deep learning model is the one-dimensional convolutional neural network (1D-CNN). Lin et al. [19] used three models, namely, FFT-DNN, FFT-LSTM, and 1D-CNN, to explore training and prediction performance. 1D-CNN is employed to automatically extract the raw vibration signal data while DNN and LSTM use the FFT feature extractor at the beginning. According to experimental research, FFT-LSTM and 1D-CNN are suggested for developing an intelligent system. Furthermore, CNN and LSTM are sometimes incorporated together to make predictions. Wang et al. [20] fused several process signals using an attentional CNN-LSTM architecture, in which CNN was utilized to extract features from process signals and the LSTM handled the sequential output of CNN. For model input, spindle current, vibration, and acoustic emission signals were selected as process signals considering the aspects of grinding wheel rotation, workpiece supporting, and material removal. Similarly, Lv et al. [21] suggested an end-to-end deep learning prediction model for improving surface roughness prediction using acceleration sensors arranged on spindles, fixtures, and metal blocks to obtain vibration signals. First, the 1D-CNN model was used to automatically extract the vibration signal features and train the data; second, an LSTM model suitable for time-series-sensitive signal training was used for the 1D-CNN training data and continued training; and finally, the fully connected classification performed predictive analysis. While a considerable body of research has been carried out on machine learning-based surface roughness prediction, much less is known about ELM applications in ultra-precision milling cases. What is more, the inputs of the majority of models are unitary, which are either cutting parameters or signal data. As a result, the novelties of this paper exist both in the application scene and in the data fusion technique that improv es model prediction accuracy.

## 3. Methodology

**Figure 1:**
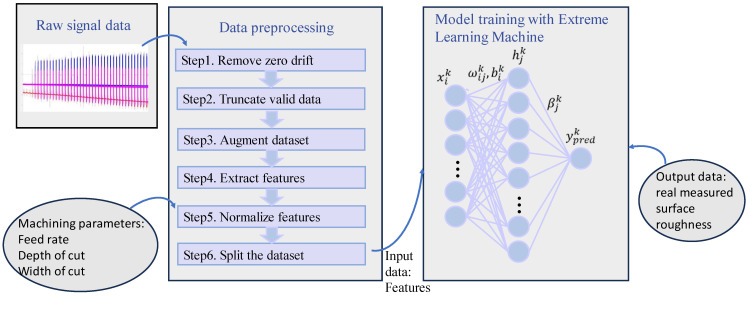
The framework of training an extreme learning machine model for surface roughness prediction in the ultra-precision milling process with the fusion of machining parameters and force signal data. Raw signal data and machining parameters are fused on a feature level in step 5, composing the final feature map as the input of ELM. The blue, red and pink raw signals shown in the small picture in the upper left corner are x-direction force signal, y direction force signal and z-direction signal respectively.

Figure 1 shows a framework for building an ELM model with data fusion for surface roughness prediction in the ultra-precision milling process. As shown in Figure 1, a detailed data preprocessing procedure is illustrated incorporating machining parameters and features from sensor data on a feature level. Specifically, machining parameters are taken as three new features and added after features extracted from sensor data in step 5 to formalize the final feature map. The inputs for the ELM model are the feature map comprising of normalized machining parameters (feed rate, depth of cut, and width of cut) and 12 time-domain features extracted from force signals, and the model output is the real-measured surface roughness. Since the raw force signal data are long, one-dimensional time series data, they should be preprocessed from step 1 to step 6. The machining parameters have only three values for each sample, so they are only preprocessed from step 5 to step 6. An ELM is chosen as the prediction model because of its fast learning speed and good fitting ability with small datasets. 

### 3.1. Extreme Learning Machine

Since the ELM approach was first proposed by Huang et al. [22] from Nanyang Technological University, it has attracted much attention, as it has been deeply studied both in its basic theory and applications. Similar to the three-layer backpropagation neural network, the ELM is a single hidden layer feedforward neural network that trains the weights between the hidden layer to the output layer in the learning stage. The difference is, normally, gradient-based algorithms are used to determine the weights and bias in the three-layer backpropagation neural network, but the ELM randomly decides the weights and biases between the input layer and the hidden layer and only computes the weights between the hidden layer and the output layer by determining Moore–Penrose directly, which can significantly save training time as parameters to be trained decrease and can be mathematically determined in one time. The number of neurons in the first layer of the ELM is the same as the number of features after data preprocessing. Therefore, only one hyperparameter, the number of neurons in the hidden layer, needs to be adjusted during the learning stage. A simple loop traversal is coded to find the best hyperparameter aimed at finding the best accuracy. 

As shown in Figure 1, the training samples are represented as $\left\{x_{i}^{k},\;y^{k}|x_{i}^{k}\in R^{D},\;y^{k}\in \;R^{m},\;i=1,\;2,\;\ldots ,\;N\right\}$, and the hidden layer is computed as shown in Equations (1)–(3).

(1) $$H\left(x\right)=\left[h^{1}\left(x\right),h^{2}\left(x\right),\ldots ,\;h^{J}\right]$$

(2) $$g\left(x\right)=\frac{1}{1+e^{-x}}$$

(3) $$h_{j}^{k}\left(x\right)=g\left(\omega_{ij}^{k}\cdot x_{i}^{k}+b_{i}^{k}\right),\;\omega_{ij}^{k}\in R^{D},\;b_{i}^{k}\in R$$

where H(x) is the output matrix in the hidden layer; $h^{j}\left(x\right)$ is the output of the $j_{\mathrm{th}}$ node in the hidden layer; g is the activation function: sigmoid; and $\omega_{ij}^{k}$ and $b_{i}^{k}$ are weights and bias in the hidden layer, which are randomly generated parameters. The output layer of the ELM is illustrated in Equation (4).

(4) $$y_{pred}^{k}=\overset{J}{\underset{j=1}{\sum }}\beta_{j}^{k}\cdot h_{j}^{k}\left(x\right)=H\left(x\right)\cdot \beta$$

where $y_{pred}^{k}$ is the predicted surface roughness value, and $\beta_{j}^{k}$ is the weight of the jth neuron in the hidden layer to the output layer. The main goal of the network is to minimize the training error between predicted surface roughness and real-measured surface roughness, as shown in Equation (5).

(5) $$min{\left(H\left(x\right)\cdot \beta -Y_{real}\right)}^{2}$$

where $Y_{real}$ is the matrix constituting the actual measured surface roughness value. In the ELM, the least square method is used to compute β, which can achieve a better performance. The solution to β is shown in Equations (6) and (7).

(6) $$\beta^{*}=H^{+}\cdot Y_{real}$$

(7) $$H^{+}={\left(H^{T}H\right)}^{-1}H^{T}$$

where $H^{+}$ is the Moore–Penrose generalized inverse matrix of H. $H^{T}$ is the transpose matrix of H. In this way, the only parameters that need to be trained can be determined. 

### 3.2. Data Preprocessing

The data preprocessing procedure is shown in the left part of Figure 1. After 6 steps of preprocessing, the raw force signal data and machining parameters can be transformed into a normalized feature map as the input of the ELM, where features are along the column, and samples are along the row. The functionality of each step is introduced below. 

Step 1. Remove zero drift. Force signal data are collected from the Kistler 3-component measuring system but encounter the zero drift problem, which means the effect where the zero reading of an instrument is modified by a change in ambient conditions. Thus, the first step of data preprocessing aims to minimize or even eliminate zero drift.

Step 2. Truncate valid data. Milling a flat surface requires tool advances and withdrawal several times since the diameter of the tool is smaller than the width of the surface to be milled. As a result, the force signals are only available when the tool is in contact with the workpiece surface, where effective force data need to be segmented from the raw signal data, which are sparse and contain many zero values. Thus, step 2 is to truncate valid data from raw signals. 

Step 3. Dataset augmentation. The paired data consist of features from sensor data and machining parameters as inputs and corresponding real-measured surface roughness as the output. Each sample is one pair of data. In sensor data collection, a high sampling frequency is set, and data are collected for several seconds in each sample. Thus, the sensor data are very long, one-dimensional time series data. Considering the complexity of conducting actual experiments resulting in the limited dataset, the dataset should be augmented to improve the prediction accuracy. Original long one-dimensional force signals are cut with a small-sized time window. However, the augmented data cut from the same force signal share the same label (real measured surface roughness) and the same combination of milling parameters. The details of this data augmentation technique have been clearly illustrated by Tieng et al. [23]. 

Step 4. Feature extraction. For force signals, 12 time-domain features are extracted for each sample after augmentation. The involved time-domain features are the peak, root mean square, crest factor, kurtosis, skewness, root mean square amplitude, margin factor, mean, pulse factor, waveform indicators, variance, and standard deviation. 

For each sample before augmentation, machining parameters are designed differently with the full-factorial experiment design method. Thus, there are 64 combinations of machining parameters. For samples after augmentation, the newly generated samples inherit the same machining parameters as their corresponding mother sample and the surface roughness from the mother sample. Therefore, the combination of machining parameters is still 64, and son samples generated from the same mother sample have the same machining parameters and surface roughness label. After step 4, three machining parameters, namely, the feed rate, depth of cut, and width of cut, are attached after the time-domain features along the column to form a new feature map. 

Step 5. Normalization. Based on the above process, a feature map, whose column is different features (time-domain features from the force signal and three machining parameters) and whose row is different samples, is formed. To improve the accuracy and integrity of the data, data normalization ensures uniformity in how the data can be utilized across the whole dataset. Z-score normalization, as shown in Equation (8), is adopted for the normalization of input data along each column in the feature map formed after step 4. The final feature map is ready to be fed as the input of the ELM model.

(8) $$x_{nf}^{j}=\frac{x_{f}^{j}-\mu^{j}}{\sigma^{j}}$$

where $x_{nf}^{j}$ is the normalized $j_{\mathrm{th}}$ feature; $x_{f}^{j}$ is the original jth feature; $\mu^{j}$ and $\sigma^{j}\;$ are the mean and standard deviation of $x_{f}^{j}$, respectively. 

Step 6. Split the training and testing datasets. The train–test split is a technique for evaluating the performance of a machine learning algorithm. Splitting your dataset is essential for an unbiased evaluation of prediction performance. The training dataset is used to fit the model and the testing dataset is used to test the model after completing the training. In this work, the training and testing datasets are divided by a ratio of 4 to 1, which means 80% of the data are for training, and 20% of the data are for testing. 

## 4. Experimental

**Figure 2:**
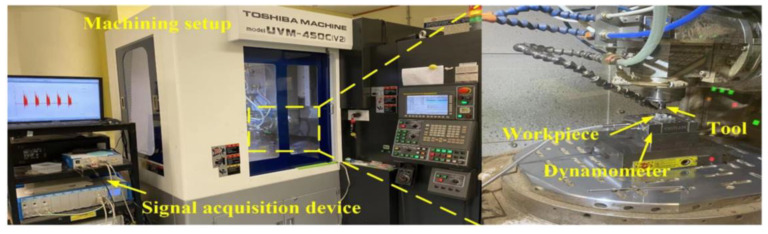
The hardware installation of the ultra-precision milling experiment.

**Figure 3:**
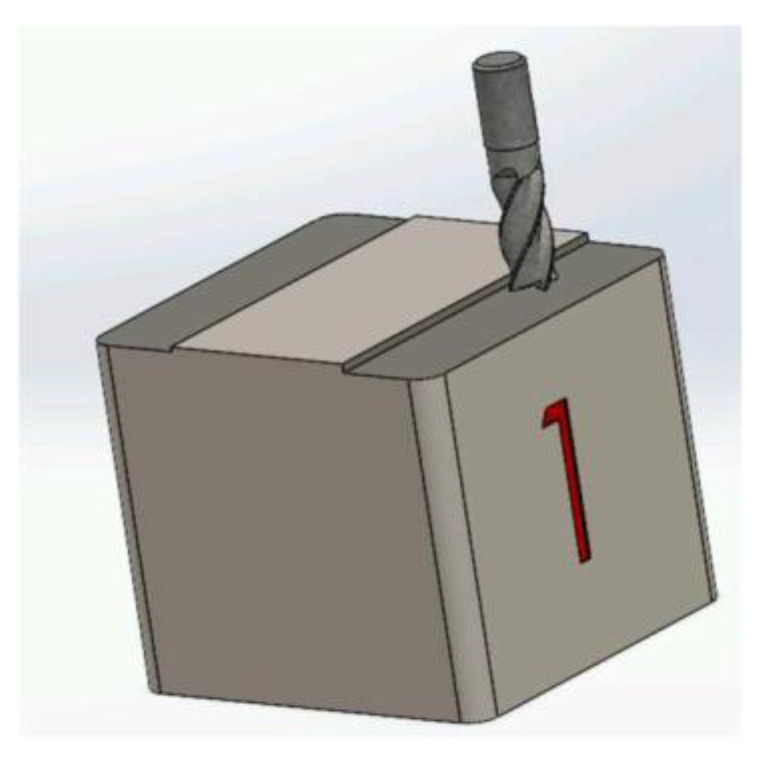
The schematic image of the milled zone on the workpiece.

To verify the effectiveness of the proposed ELM with a data fusion framework, a real ultra-precision milling experiment is designed and conducted on a Toshiba UVM five-axis micro-milling machine to collect the dataset for model training. The hardware configuration and setup for the milling experiment are presented in Figure 2. To see the structures of milled surface roughness clearly, Figure 3 provides a schematic image of the machined workpiece. In order to increase the utilization of the workpieces, two combinations of milling parameters are milled on the same plane. To distinguish different trials, the trial numbers are lettered on both sides. Each workpiece is cubic with a length of 8 mm, a processing area of 2 mm on both sides, and a margin of 4 mm. 

Three primary machining parameters, namely, the feed rate, depth of cut, and width of cut, are widely recognized as the crucial controllable factors influencing surface roughness during the face milling process [24]. The spindle speed is set to a fixed parameter (60,000 rpm) in this experiment, as the minimum spindle speed of the high-speed air float spindle used for the Toshiba high-precision micro-milling machine is 60,000 rpm, and the spindle speed for ultra-precision milling is already a relatively optimal value. A full factorial experiment is applied to design different machining conditions. Based on the capabilities of the ultra-precision milling machine and the desired range of value commonly used in the industry, each parameter is set to four different levels, providing a total of 64 experiment runs for each parameter combination. The four levels of feed rate are 200 mm/min, 400 mm/min, 600 mm/min, and 800 mm/min. The four levels of depth of cut are set to 30 mm, 60 mm, 90 mm, and 120 mm. Moreover, 5 mm, 100 mm, 150 mm, and 200 mm are the four setting values for the width of the cut. Table 1 shows the levels of each parameter used in the experiment. In total, 64 groups of milling experiments are conducted under different combinations of control parameters. 

The CrTiAlN-coated cemented carbide and micro-milling tool (model numer: MXH230, manufacturer: NS Tool from Nankowu city, Japan) is used to milled the specimens. In order to minimize the impact of tool wear, each tool is replaced with a new one after collecting eight original samples during the actual experiment. The dynamometer (Kistler 9256C1, manufacturer: Kistler from Winterthur, Switzerland) is fixed under the workpiece, and the charge amplifier (Kistler multichannel charge amplifier 5080, manufacturer: Kistler from Winterthur, Switzerland) and PC software (Dynoware version 3.0.9.0) are used to measure force in the three orthogonal directions along the *X*-axis, *Y*-axis, and *Z*-axis. The sampling ratio for the cutting force measurement is set to 20 kHz. In the experiment, the specimen is made of die steel and the milling tool is made of cemented carbide. 

**Figure 4:**
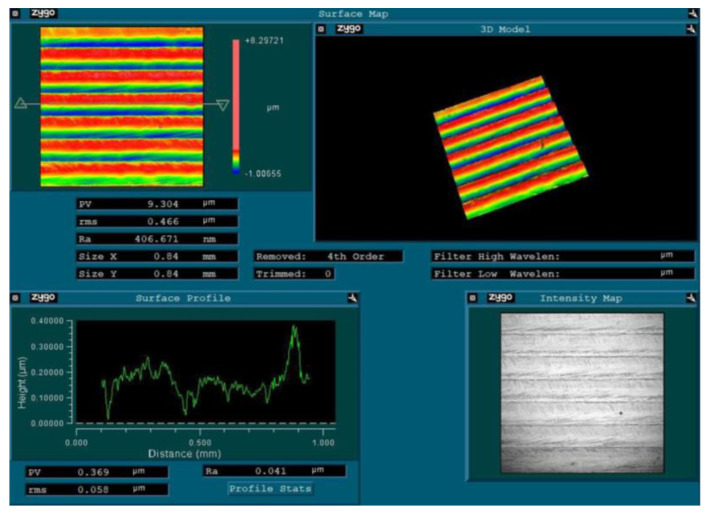
Surface map of the workpiece.

After the milling process, surface roughness is measured with a ZYGO NEXVIEW (manufacturer: Zygo Corporation from Middlefield, Connecticut, USA) white light interferometer. The interferometer uses white light to scan the surface of the workpiece and collect data on the height and position of each point. The roughness measurement selected for this study is arithmetic roughness, $R_{a}$, which represents the average distance between the highest and lowest points of the surface texture profile over a specified sampling length. Once the measurement is complete, the data collected by the interferometer will be analyzed using the NexView software (version: Mx 9.0.0.20) to determine the $R_{a}$ value, as shown in Figure 4. The analysis results are saved and exported for further analysis.

## 5. Results and Discussion

The data adopted in this section is acquired from the above ultra-precision milling experiment. From the Taguchi experiment design, there are a total of 64 sets of samples with different combinations of machining parameters. After data augmentation, the dataset is enlarged to 960 samples, where 80% (768) of the samples are randomly selected for training and 20% (192) of the samples are for testing. To prove the fast learning speed and high prediction accuracy of our proposed ELM with a data fusion method, many state-of-the-art machine learning methods are applied in this case, with training and testing on the same dataset. As declared in our review of recent related works, four machine learning models, namely, the long short-term memory network (LSTM), the one-dimensional convolutional neural network (1DCNN), the integrated convolutional neural network and long short-term memory (CNN-LSTM), and the backpropagation neural network (BPNN) are chosen to prove the effectiveness of our proposed ELM with data fusion approach.

Firstly, five machine learning models are compared with each other in terms of mean absolute percentage error (MAPE), while only the features from the force signal are used as the model input. For the 1D-CNN and CNN-LSTM, data preprocessing for force signals only has five steps, which excludes the feature extraction (step 4) in Figure 1, as these two machine learning methods have an automatic feature extraction ability. For the LSTM, BPNN, and ELM, force signal preprocessing follows the six steps indicated in Figure 1. Besides evaluating the accuracy of the model, training time also should be taken into consideration for the application of the future online prediction, which is significantly affected by the model adjustment time (retraining time). Therefore, training time attracts attention in particular while performing different methods. 

**Figure 5:**
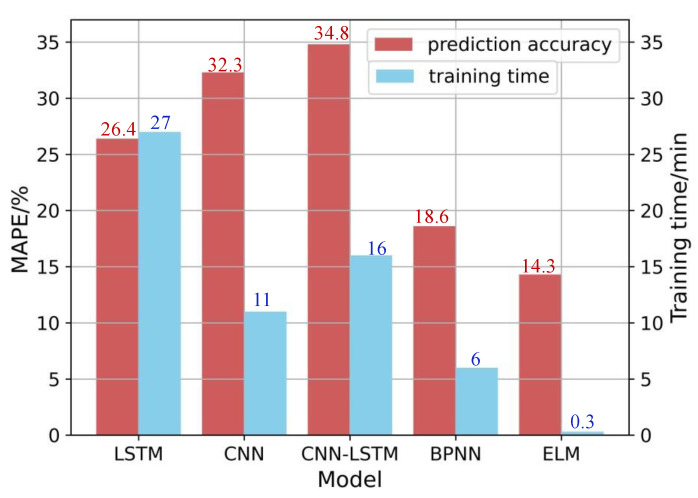
Comparison of different models’ performance in prediction accuracy and training time for surface roughness prediction in ultra-precision milling.

**Figure 6:**
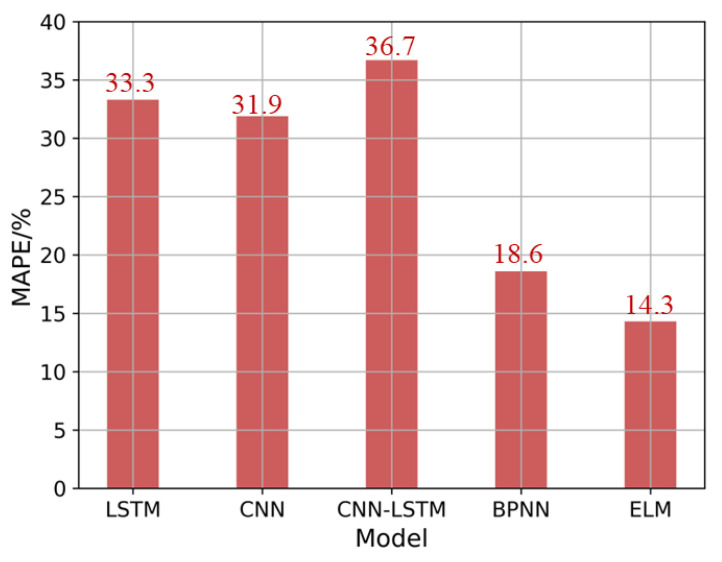
Comparison of model training efficiency while training for 10 min.

A comparison of the models’ performance regarding prediction accuracy and the total training time in the ultra-precision milling surface roughness prediction case is shown in Figure 5. The red bar represents the model prediction accuracy evaluated by the MAPE. The smaller the MAPE, the better the prediction accuracy. The blue bar represents the total time required to complete training. The CNN-LSTM and CNN have the worst performance, with MAPEs over 30%. This is probably because although data augmentation increases the size of the dataset, the richness and diversity of the dataset have no significant improvement. For the CNN-LSTM and CNN, it is hard to leverage their automatic feature extraction power under the dataset where data diversity is not rich enough. In terms of total training time, the ELM reaches its best accuracy, taking no more than 1 min, while the LSTM takes the longest time to converge. To compare the model efficiency intuitively, Figure 6 shows a comparison of model-training efficiency after training for 10 min. As the ELM and BPNN have finished the whole training process within 10 min, their final prediction accuracy is shown in Figure 6. After training for 10 min, the prediction accuracy of the CNN, CNN-LSTM, and LSTM is still larger than 30%. Especially for LSTM-related approaches, training efficiency is lower than other approaches. The reason LSTM-related methods have longer training time than other methods may be that Tensorflow 2.0 does not optimize the LSTM implementation for batch processing, resulting in slow performance over time. The LSTM, BPNN, and ELM use handmade features as model inputs to have better prediction accuracy, which is consistent with existing knowledge showing that handcrafted features as model inputs usually perform better than learned features relying on a large number of labels for small datasets [25]. Among all the chosen methods, the BPNN and ELM have relatively high performance in prediction accuracy. The prediction accuracy of the BPNN is 18.6%, which is quite close to the prediction accuracy of the ELM with a MAPE of 14.3%. However, the BPNN takes about 6 min to converge, and the ELM spends no more than 1 min to outperform the other four models. Therefore, the ELM outperforms other approaches both in prediction accuracy and efficiency.

**Figure 7:**
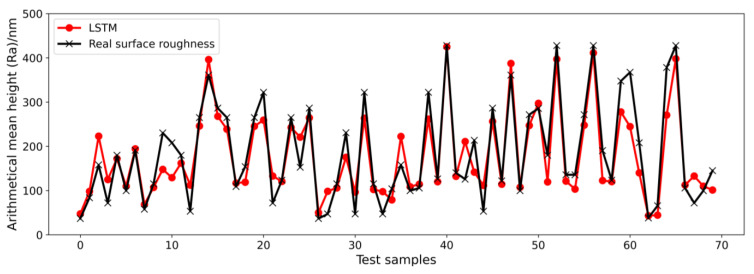
Prediction results of the LSTM on the test dataset with a 26.4% MAPE with only force signals as the model input.

**Figure 8:**
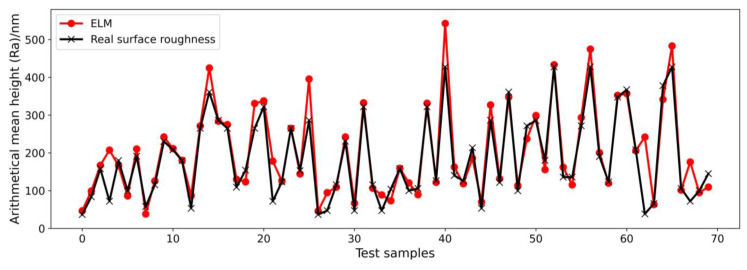
Prediction results of the ELM on the test dataset with a 14.3% MAPE with only force signals as the model input.

**Figure 9:**
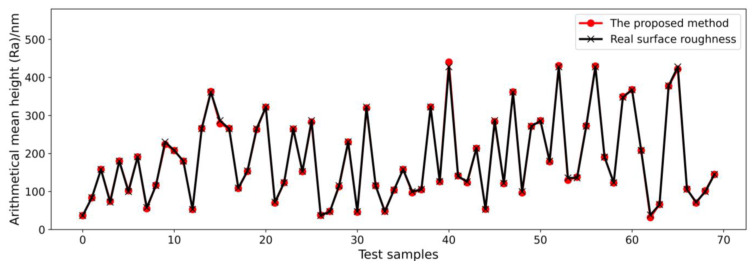
Prediction results of the proposed ELM with a data fusion method on test dataset with a 1.6% MAPE.

To obtain a glimpse of the good performance of the ELM more clearly, the prediction results showing a 26.4% MAPE for the LSTM and prediction results showing a 14.3% MAPE for the ELM are shown in Figure 7 and Figure 8, respectively. The black line connects the points from real-measured surface roughness, and the red line connects the points from the model prediction results. The MAPE is computed based on 192 samples (the whole test dataset). For the convenience of visualization, only the first 70 samples in the test datasets are shown. In Figure 7, some red points cannot overlap with the black points in many points, such as points around test samples of 10, 20, 60, and 70, which means the predicted surface roughness deviates from the real-measured surface roughness by a lot. As shown in Figure 8, the ELM prediction results are generally consistent with the changing trend in the real-measured surface roughness, except for some points around test samples of 65. However, a 14.3% MAPE is not good enough. Considering that only force signal data were used as the model input until now, machining parameters should also be taken into account, as they directly influence the machining surface roughness. As a result, we propose a data fusion method that fuses machining parameters and features from force signals on a feature level for the ELM model to train the ultra-precision milling surface roughness prediction model. Figure 9 illustrates how our proposed ELM with a data fusion model improves prediction accuracy dramatically from a MAPE of 14.3% to a MAPE of 1.6%. As shown in Figure 9, the prediction results from the proposed method agree well with the measured surface roughness results, which indicates that the proposed ELM with a data fusion method is suitable for surface roughness prediction in ultra-precision milling. Moreover, the proposed ELM method is very effective and efficient, which only requires a training time of 18 s, while other methods take over 10 min. The efficiency of the prediction algorithm is important for real-time prediction and parameter adaptation. The results prove that our proposed ELM method outperforms other state-of-the-art methods in terms of prediction accuracy and efficiency. A key point that needs to be emphasized is that this paper only constructed an offline ultra-precision milling surface roughness prediction model, which means the data used for model training came from a previous experiment but not the ongoing machining process. In online application, the accuracy will remain great if the milling condition is very similar to the former design experiment and there is no other significant tool wear to change the input data distribution. In other words, the generalization performance depends on whether the milling condition changes a lot. If there is significant tool wear, or unexpected vibration occurs, the model accuracy may deteriorate.

## 6. Conclusions

An extreme learning machine (ELM) approach with data fusion of machining parameters and force signal features is presented for surface roughness prediction in ultra-precision milling. When only force signal data are the model input, compared with other state-of-the-art methods (LSTM, CNN, CNN-LSTM, and BPNN), testing on the real dataset collected from a fully factorially designed ultra-precision milling experiment proves that the ELM outperforms other state-of-the-art methods in terms of prediction accuracy with a MAPE of 14.3%. To further improve the prediction accuracy, a feature-level data fusion technique is applied to the ELM, which dramatically improves the prediction accuracy from a MAPE of 14.3% to a MAPE of 1.6%, which makes the prediction results agree well with the real-measured surface roughness. At the same time, the proposed ELM with data fusion is highly efficient and takes only 18 s for model training. This paper only provides an offline ultra-precision milling surface roughness prediction model. However, for future online applications, a model refreshment mechanism should be developed to avoid prediction accuracy drift and maintain the model prediction accuracy.

## Figures and Tables

**Table 1:** Cutting parameters of the experiment.

Control Parameter	Units	Levels			
		1	2	3	4
Feed rate	mm/min	200	400	600	800
Depth of cut	mm	0.03	0.06	0.09	0.12
Width of cut	mm	0.05	0.1	0.15	0.2

## Data Availability

The original dataset is unavailable for privacy reasons, but you can archive the source code at the time of publication: https://zenodo.org/records/10032802.
